# U-shaped association of resting heart rate with cognitive decline

**DOI:** 10.1093/braincomms/fcaf413

**Published:** 2025-10-21

**Authors:** Eugene S J Tan, Ming Ann Sim, Lucia Li, Anqi Toh, Eddie J Y Chong, Siew Pang Chan, Cheuk Ni Kan, Xin Tong Tan, Jiangbo Cui, Saima Hilal, Joyce R Chong, Mitchell K P Lai, Narayanaswamy Venketasubramanian, Boon Yeow Tan, A Mark Richards, Lieng-Hsi Ling, Christopher L H Chen

**Affiliations:** Department of Cardiology, National University Heart Centre, Singapore 119074, Singapore; Department of Medicine, Yong Loo Lin School of Medicine, National University of Singapore, Singapore 117600, Singapore; Department of Medicine, Yong Loo Lin School of Medicine, National University of Singapore, Singapore 117600, Singapore; Memory Aging and Cognition Center, National University Health System, Singapore 117599, Singapore; Department of Intensive Care Medicine, Ng Teng Fong General Hospital, Singapore 609606, Singapore; Memory Aging and Cognition Center, National University Health System, Singapore 117599, Singapore; School of Clinical Medicine, Cambridge University, Cambridge CB2 0SP, United Kingdom; Memory Aging and Cognition Center, National University Health System, Singapore 117599, Singapore; Memory Aging and Cognition Center, National University Health System, Singapore 117599, Singapore; Department of Cardiology, National University Heart Centre, Singapore 119074, Singapore; Memory Aging and Cognition Center, National University Health System, Singapore 117599, Singapore; Memory Aging and Cognition Center, National University Health System, Singapore 117599, Singapore; Memory Aging and Cognition Center, National University Health System, Singapore 117599, Singapore; Department of Pharmacology, Yong Loo Lin School of Medicine, National University of Singapore, Singapore 117600, Singapore; Memory Aging and Cognition Center, National University Health System, Singapore 117599, Singapore; Department of Pharmacology, Yong Loo Lin School of Medicine, National University of Singapore, Singapore 117600, Singapore; Saw Swee Hock School of Public Health, National University of Singapore, Singapore 117549, Singapore; Memory Aging and Cognition Center, National University Health System, Singapore 117599, Singapore; Department of Pharmacology, Yong Loo Lin School of Medicine, National University of Singapore, Singapore 117600, Singapore; Memory Aging and Cognition Center, National University Health System, Singapore 117599, Singapore; Department of Pharmacology, Yong Loo Lin School of Medicine, National University of Singapore, Singapore 117600, Singapore; Raffles Neuroscience Centre, Singapore 118700, Singapore; St. Luke’s Hospital, Singapore 659674, Singapore; Department of Cardiology, National University Heart Centre, Singapore 119074, Singapore; Department of Medicine, Yong Loo Lin School of Medicine, National University of Singapore, Singapore 117600, Singapore; Christchurch Heart Institute, University of Otago, Christchurch 8011, New Zealand; Department of Cardiology, National University Heart Centre, Singapore 119074, Singapore; Department of Medicine, Yong Loo Lin School of Medicine, National University of Singapore, Singapore 117600, Singapore; Memory Aging and Cognition Center, National University Health System, Singapore 117599, Singapore; Department of Pharmacology, Yong Loo Lin School of Medicine, National University of Singapore, Singapore 117600, Singapore

**Keywords:** bradycardia, heart rate, cognitive decline, biomarkers, cerebrovascular disease

## Abstract

Reports of associations between bradycardia and elevated resting heart rate with cognitive decline are inconsistent, and the underlying mechanisms unclear. Resting heart rate was recorded in a prospective cohort of memory clinic subjects, and the associations of resting heart rate with cross-sectional cognition, brain magnetic resonance imaging and circulating biomarkers (pTau-181 and neurofilament light chain), and longitudinal cognitive decline were investigated. Subjects with atrial fibrillation were excluded. Among 643 subjects (mean age 72.8 ± 8.0 years, 57% female, 39% dementia), 35% had resting heart rate between 60 and 69 bpm, 23% had bradycardia (<60 bpm) and 42% had elevated resting heart rate (≥70 bpm). Compared to 60–69 bpm, both bradycardia (<60 bpm) and elevated resting heart rate ≥70 bpm were associated with worse baseline global cognition (*P* < 0.05), and elevated resting heart rate additionally with worse Clinical Dementia Rating-Sum of boxes scores (*P* < 0.05) and executive function on multivariable adjustment (*P* = 0.001). Longitudinally, resting heart rate ≥70 bpm was associated with accelerated global cognitive decline, and bradycardia with accelerated functional decline (*P*_interaction_ < 0.05). Regarding neuroimaging and circulating biomarkers, bradycardia was associated with reduced grey matter volume, and higher levels of circulating pTau-181 and neurofilament light chain when compared to resting heart rate 60–69 bpm (*P* < 0.05). Conversely, elevated resting heart rate ≥70 bpm was associated with higher burden of cortical infarcts, cerebral microbleeds and lacunes compared to resting heart rate 60–69 bpm, and higher white matter hyperintensity volume ratio and cerebral microbleeds compared to bradycardia (*P* < 0.05). Resting heart rate displayed a U-shaped association with cognitive impairment and structural cerebral abnormalities, and may be underpinned by distinct mechanisms. Further study of the mechanisms underlying resting heart rate and cognitive trajectories are warranted. **Trial registration:** National Healthcare Group Domain Specific Review Board 2018/01098 and 2010/00017

## Teaser

Tan *et al*. report a U-shaped association between resting heart rate and cognitive decline. Distinct associations with neuroimaging and circulating biomarkers suggest neurodegenerative and vascular-mediated mechanisms in bradycardia and elevated resting heart rate, respectively. Further mechanistic studies into resting heart rate-related cognitive trajectories are needed.

## Graphical Abstract

**Graphical Abstract:**
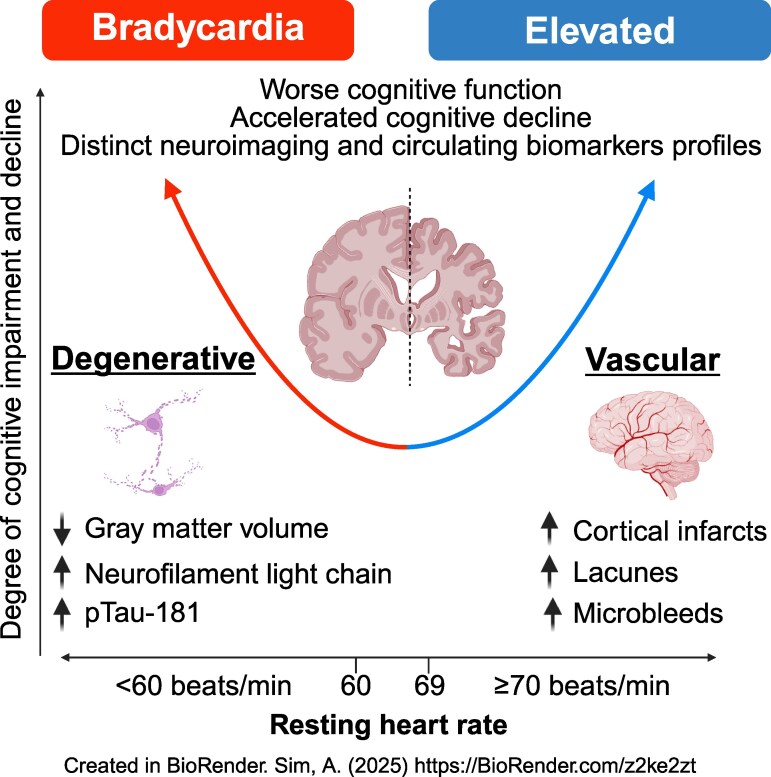

## Introduction

Dementia is a debilitating disease associated with impaired quality of life and functional disturbances.^1^ With an aging population, the estimated global prevalence of dementia is expected to increase to 153 million individuals by 2050.^2^ In the absence of definitive treatments, management must focus on the prevention and management of modifiable risk factors such as obesity, hypertension, diabetes mellitus, smoking and alcohol misuse, among others identified in the 2024 update to the Lancet Commission.^3^ Apart from these risk markers, resting heart rate (RHR) has recently emerged as a potential biomarker of cognitive impairment.

In several large cohort studies, elevated RHR ≥70 bpm when compared to lower RHR, was significantly associated with increased risk of dementia and accelerated cognitive decline based on reduced scores on the Mini Mental State Examination (MMSE).^4-8^ Elevated RHR was further shown to be associated with greater ischaemic brain lesion volumes, and circulating biomarkers of inflammation and endothelial injury.^7,9^ Conversely, the association of bradycardia with cognitive decline is less certain. Despite a reported J-shaped association between RHR and incident dementia^5,7^ and reports of improved cognitive function after pacemaker implantation for symptomatic bradycardia,^10,11^ the association of low RHR with cognitive decline and incident dementia has never been substantiated, with some evidence suggesting instead an association with better cognition in the post-ischaemic stroke setting.^4,5,8^ The use of insensitive cognitive assessment metrics and heterogeneous study populations may account for some of these inconsistencies. However, misfolded protein aggregates such as amyloid-β (Aß) and tau protein can accumulate in organs other than the brain^12,13^ potentially disrupting electrical impulse propagation resulting in bradyarrhythmia and conduction abnormalities.^14^ This raises the hypothesis that a proteinopathy could underlie the association of low RHR and cognitive impairment. There is, therefore, a need to better characterize the longitudinal trajectory of cognitive function associated with RHR, and investigate underlying mechanisms with comprehensive cognitive assessments, circulating and neuroimaging biomarkers.

We hypothesize that the relationship between RHR and cognitive decline is non-linear, and subserved by different pathophysiological mechanisms. Accordingly, the aims of this study are to determine, in a cohort of memory clinic subjects, associations of bradycardia (<60 bpm) and/or elevated RHR (≥70 bpm), compared to RHR 60–69 bpm, with (i) the trajectory of cognitive and functional decline (ii) cerebrovascular disease (CeVD) on neuroimaging and (iii) circulating biomarkers.

## Materials and methods

### Study population

Subjects included in this cohort were memory clinic patients recruited from two public health institutions in Singapore and followed for up to 5 years in a prospective, longitudinal study of cognitive impairment.^15^ All subjects were ≥50 years and provided written informed consent. Those with a history of major psychiatric illness, substance abuse, traumatic brain injury, brain tumours, multiple sclerosis and epilepsy were excluded from analysis. Subjects with a history of atrial fibrillation (AF) were also excluded due to the known adverse effects of AF on cognitive function.^16^ Ethics approval was obtained from the local institutional review board and the study complied with the Declaration of Helsinki.

### Clinical variables

Data on age, sex, body mass index (BMI), years of education and clinical comorbidities including hypertension, diabetes, rate-limiting medications (beta-blockers, non-dihydropyridine calcium channel blockers, digoxin) and cognitive enhancers (donepezil, rivastigmine, memantine) were obtained from detailed questionnaires at recruitment and verified on electronic health records. Apolipoprotein E4 (ApoE4) genotyping was performed, with ApoE4 carrier status defined according to presence of the ApoE4 allele. At baseline study visit, blood pressure and heart rate measurements were made with an automated blood pressure machine after subjects have rested for 10 min. The second of two measurements made was recorded. Subjects were then classified into three groups according to category of RHR at baseline: <60, 60–69, or ≥70 bpm.

### Neurocognitive assessment and diagnosis

Annual cognitive assessments by trained neuropsychologists were performed via the mini mental state examination (MMSE), Montreal Cognitive Assessment (MoCA) and a locally validated neuropsychological test battery as described.^17^ Individual cognitive domains assessed include memory, visuoconstruction, visuomotor speed, attention, language and executive function (protocol in Supplementary Table 1). A composite global cognitive score was averaged from these domains, and standardized z-scores (mean ± standard deviation [SD]) were calculated. Neurocognitive diagnoses were discussed at consensus meetings between clinicians and neuropsychologists after review of neurocognitive and neuroimaging data. On longitudinal follow-up global cognitive z-scores were calculated in a similar fashion, using the means and standard deviations of the control group evaluated at baseline.

Subjects were classified as no cognitive impairment (NCI), cognitive impairment without dementia (CIND) in the presence of impairment in ≥1 domain (defined as <1.5SD below education level-adjusted cut-off for each test in ≥50% of tests in each domain) but not meeting criteria for dementia, and dementia, defined according to the Diagnostic and Statistical Manual of Mental Disorders-IV edition.^18^ The Clinical Dementia Rating Scale Sum of Boxes (CDR-SB) score was calculated at baseline and at each follow-up visit to indicate severity and rate of change in cognitive and functional deficit.

### Neuroimaging findings

Magnetic resonance imaging (MRI) brain scans were performed at baseline according to standardized protocols (Supplementary Table 2). Documented neuroimaging markers included grey matter volume (GMV), white matter (WMV), hippocampal and white matter hyperintensity (WMH) to total brain volume ratio, white matter hyperintensity ratio, cortical infarcts, cerebral microbleeds (Brain Observer Microbleed Scale) and lacunes (3–15 mm). All were graded by a single observer (S.H.) with intra-rater agreement of ≥0.8.

### Circulating biomarkers

Venous samples obtained at baseline were drawn into EDTA tubes and centrifuged with separation of plasma and stored at −80°C prior to assay. Plasma tau phosphorylated at threonine-181 (pTau-181), neurofilament light chains (NFL) and glial fibrillary acidic protein (GFAP) were measured with the SIMOA platform (Quanterix Corp., Billerica, MA, USA) with <5% intra- and inter-assay coefficient of variance (CoV) for pTau-181 and NFL previously described,^19^ and <15% for GFAP. In exploratory analyses, circulating pTau-217 protein levels were measured with the Nucleic acid-Linked Immuno-Sandwich Assay (NULISA) (NULISASeq™ CNS Disease Panel 120, Alamar Biosciences Inc., Fremont, CA) and normalized into NULISA Protein Quantification (NPQ) units for comparison between RHR groups.^20^ Prior to analysis, the pTau-217 NPQ units were validated against quantitative pTau-181 levels measured with the SIMOA platform, and found to have a high level of correlation (r > 0.8).

### Statistical analysis

Baseline characteristics were expressed as mean ± standard deviation or median (inter-quartile range), and compared between groups with the Kruskal–Wallis test or Wilcoxon Rank Sum test (continuous) and the χ^2^ test (categorical). The cross-sectional associations of RHR with baseline cognition (global and individual domain Z-scores) and CDR-SB were determined by linear regression and adjusted for baseline demographics and clinical comorbidities in stepwise fashion for age, sex and education, followed additionally by ApoE4 status, systolic blood pressure (SBP), history of hypertension, diabetes mellitus and rate-limiting medications. Linear mixed-effect regression models based on two-level random-intercept models with restricted maximum likelihood technique were constructed to examine the associations of RHR with the rate of cognitive and functional decline previously described,^15^ adjusting first for baseline demographics (age, sex, education), followed by clinical comorbidities (ApoE4 status, SBP, hypertension, diabetes, rate-limiting medications), baseline MMSE score and cognitive enhancers in the same stepwise manner. Longitudinal visit data were nested within subjects and the intercept was allowed to vary. Significant interaction with time (*P*_interaction_ <0.05) indicated a significant difference in rate of decline for global cognition Z-score and CDR-SB between RHR categories, while a *P*_interaction_ of <0.0083 was used for domain-specific cognition to correct for multiple testing. Cross-sectional associations of RHR with CeVD counts on neuroimaging were ascertained by negative binomial regression in view of evidence of overdispersion based on deviance residuals, and with circulating biomarkers (log-transformed to approximate normal distribution) in robust regression estimated with iteratively reweighted least squares, respectively. The associations of RHR with cognitive and functional decline, circulating and neuroimaging were repeated in sensitivity analyses among only subjects without dementia at baseline. Subjects with missing data were excluded from analyses. Statistical analyses were performed with STATA MP v16 at 5% level of significance.

## Results

### Comparison of baseline characteristics

**Figure 1:**
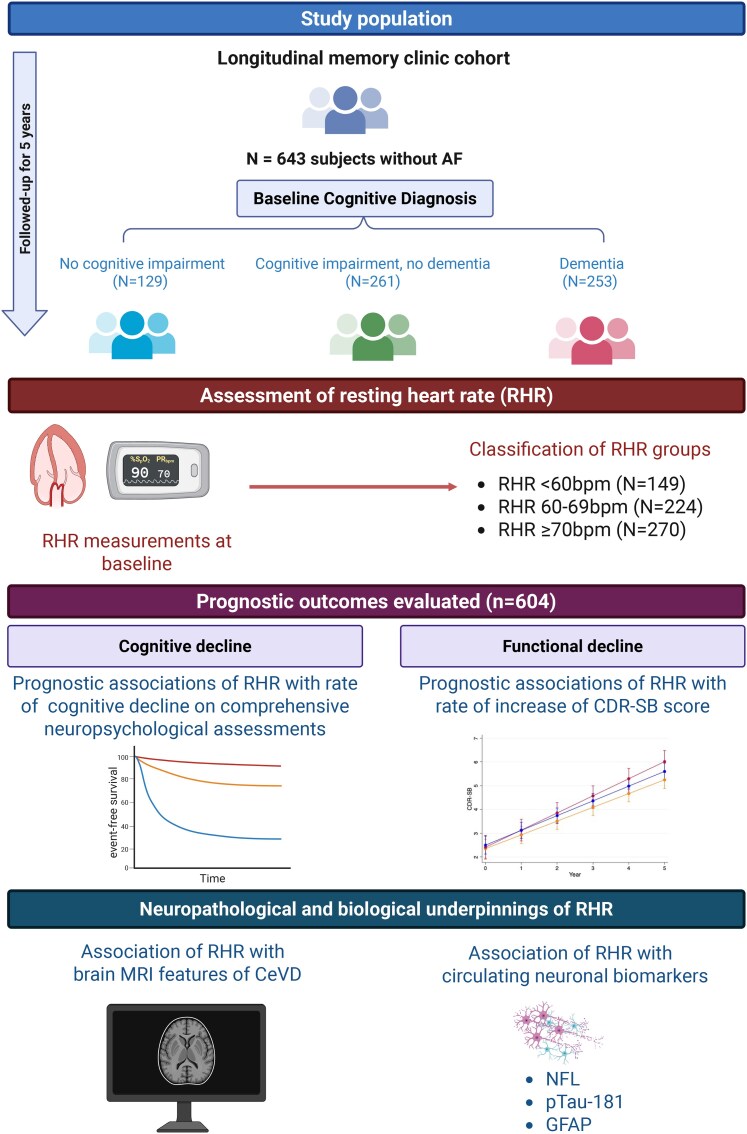
**Study design and subject inclusion.** Of 643 subjects from this memory clinic cohort with without AF, RHR was categorized as bradycardia (<60 bpm), 60–69 bpm (reference) and ≥70 bpm (elevated). Clinical outcomes assessed include the rate of cognitive and functional decline. Mechanisms underpinning associations of RHR with cognitive dysfunction were investigated with neuroimaging markers of CeVD and circulating neuronal biomarkers. (*Created in BioRender. Sim, A. (2025)*  https://BioRender.com/e80×756). AF, atrial fibrillation; CeVD, cerebrovascular disease; CDR-SB, clinical dementia rating—sum of boxes; GFAP, glial fibrillary acidic protein; MRI, magnetic resonance imaging; NFL, neurofilament light chain; RHR, resting heart rate.

Among 695 subjects from the memory clinic cohort with available RHR measurements, 643 subjects without AF were included in this study (mean age 72.8 ± 8.0 years, 57% female), of which 129 (20%) had NCI, 261 (41%) had CIND and 253 (39%) had dementia (study design shown in Fig. 1). Of these subjects, 224 (35%) had RHR between 60 and 69 bpm, 149 (23%) had bradycardia (RHR <60 bpm) and 270 (42%) had RHR ≥70 bpm. Compared to RHR 60–69 bpm, subjects with RHR ≥70 bpm had a higher prevalence of diabetes mellitus (41% versus 32%) and less frequent use of rate-limiting agents (13% versus 27%), while those with bradycardia had more frequent use of rate-limiting medications (40% versus 27%) (all *P* < 0.05; Table 1). Compared to RHR ≥ 70 bpm, bradycardia RHR ≥70 bpm had statistically higher but clinically insignificant diastolic blood pressure (*P* < 0.05).

**Table 1:** Comparison of baseline characteristics between RHR groups

	RHR 60–69 bpm	RHR < 60 bpm	RHR ≥ 70 bpm	p1	p2	p3	p4
*N*	224 (35)	149 (23)	270 (42)				
Comorbidities							
Age, years	72.8 ± 7.7	72.9 ± 8.2	72.6 ± 8.1	0.898	0.886	0.645	0.804
Female (%)	135 (60)	81 (54)	152 (56)	0.486	0.258	0.373	0.703
BMI, kg/m^2^	23.8 ± 3.8	23.8 ± 4.3	23.9 ± 3.8	0.857	0.601	0.975	0.633
ApoE4 status (%)	68 (31)	45 (31)	80 (30)	0.954	0.986	0.779	0.819
Education, years	6.6 ± 4.8	7.2 ± 5.5	6.9 ± 5.0	0.658	0.397	0.474	0.835
Systolic blood pressure, mmHg	144.4 ± 19.7	143.0 ± 20.4	142.7 ± 18.2	0.645	0.940	0.389	0.495
Diastolic blood pressure, mmHg	72.8 ± 7.7	72.9 ± 8.2	72.6 ± 8.1	0.017	0.257	0.070	0.006
Hypertension (%)	159 (71)	109 (74)	186 (70)	0.692	0.575	0.750	0.391
Diabetes mellitus (%)	71 (32)	47 (32)	111 (41)	0.047	0.975	0.031	0.053
Hyperlipidaemia (%)	163 (73)	108 (72)	196 (73)	0.989	0.952	0.927	0.886
Smoker (%)	60 (27)	43 (29)	68 (25)	0.715	0.661	0.686	0.415
Ischaemic heart disease (%)	26 (12)	20 (13)	22 (8)	0.201	0.601	0.196	0.085
Rate-limiting medications	64 (29)	62 (42)	38 (14)	<0.001	0.009	<0.001	<0.001
Beta-blockers (%)	64 (29)	61 (41)	36 (13)				
CCB (%)	0 (0)	1 (1)	2 (1)				
Digoxin (%)	1 (0.5)	0 (0)	0 (0)				
Cognitive diagnosis				0.622	0.533	0.387	0.689
NCI (%)	48 (21)	32 (21)	49 (18)				
CIND (%)	96 (43)	56 (38)	109 (40)				
Dementia (%)	80 (36)	61 (41)	112 (42)				
Cognitive enhancers (%)	60 (27)	42 (28)	77 (29)	0.907	0.766	0.668	0.943
Donepezil (%)	54 (24)	35 (23)	61 (23)				
Rivastigmine (%)	6 (3)	4 (3)	8 (3)				
Memantine (%)	3 (1)	3 (2)	11 (4)				
Circulating biomarkers							
pTau-181^a^, pg/ml (*n* = 498)	2.4 (1.6, 3.7)	3.0 (2.1, 4.5)	2.4 (1.7, 3.4)	<0.001	0.001	0.967	<0.001
NFL^a^, pg/ml (*n* = 497)	20.7 (15.9, 35.1)	25.4 (18.7, 40.5)	24.0 (15.6, 40.0)	0.061	0.016	0.149	0.333
GFAP^a^, pg/ml (*n* = 479)	264.6 (158.7, 438.4)	259.0 (145.4, 458.7)	280.7 (154.8, 454.6)	0.981	0.880	0.857	0.980

p1: *P-*value for comparison of baseline characteristics across all 3 RHR groups by Kruskal–Wallis test or χ^2^ test.

p2: *P-*value for comparison of baseline characteristics by Wilcoxon Rank Sum test or χ^2^ test between RHR <60 bpm versus 60–69 bpm.

p3: *P-*value for comparison of baseline characteristics by Wilcoxon Rank Sum test or χ^2^ test between RHR ≥70 bpm versus 60–69 bpm.

p4: *P-*value for comparison of baseline characteristics by Wilcoxon Rank Sum test or χ^2^ test between RHR ≥70 bpm versus <60 bpm.

ApoE4, apolipoprotein E4; BMI, body mass index; CCB, calcium-channel blockers; CDR-SB, clinical dementia rating—sum of boxes; CIND, cognitive impairment no dementia; NCI, no cognitive impairment; NFL, neurofilament light chain; RHR, resting heart rate.

^a^Expressed as median (inter-quartile range).

### Cross-sectional association of RHR with cognitive function

Compared to RHR 60–69 bpm, both bradycardia (ß −0.56, 95% confidence interval [CI] −1.03, −0.09, *P* = 0.020) and RHR ≥70 bpm (ß −0.51, 95% CI −0.92, −0.11, *P* = 0.013) were associated with significantly worse global cognition on comprehensive neuropsychological assessment after full adjustment in multivariable models for age, sex, education, ApoE4 status, SBP, history of hypertension, diabetes mellitus and rate-limiting medications (*P* < 0.05, Supplementary Table 3). With respect to individual cognitive domains, only RHR ≥70 bpm remained significantly associated with worse executive function in full multivariable analyses after correction for multiple testing (ß −0.59, 95% CI −0.94, −0.24, *P* = 0.001). The association of bradycardia (adjusted for age, sex, education) with worse executive function was attenuated after further adjustment for clinical comorbidities and rate-limiting medications (*P* > 0.0083 for multiple testing).

With regard to baseline functional status (CDR-SB), RHR ≥70 bpm remained significantly associated with higher CDR-SB scores when adjusted fully for patient demographics, clinical comorbidities and rate-limiting medications (ß 0.91, 95% CI 0.22, 1.60, *P* = 0.010). However, the significant association of bradycardia (adjusted for age, sex, education) with higher CDR-SB scores was attenuated after further adjustment for clinical comorbidities and rate-limiting medications (*P* = 0.071). Cross-sectional associations with global cognition and functional deficit did not differ between bradycardia and RHR ≥70 bpm.

### Longitudinal association of RHR with rate of cognitive and functional decline

**Figure 2:**
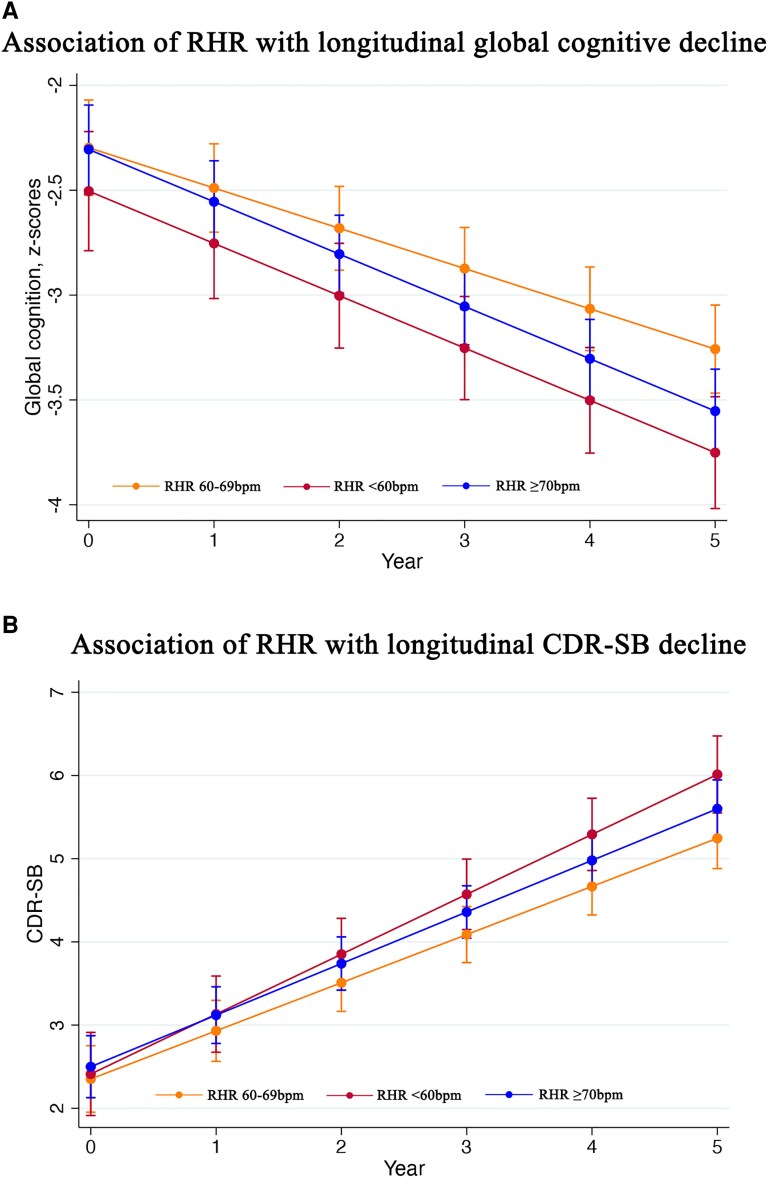
**Association of RHR with decline in global cognition and functional status.** Among subjects (*N* = 604) with long-term follow-up over 5 years, (**A**) elevated RHR ≥70 bpm was associated with accelerated global cognitive decline, and (**B**) bradycardia was associated with accelerated decline in CDR-SB scores, compared to RHR 60–69 bpm, in linear mixed-effects regression analyses. The association of RHR with longitudinal decline in global cognition and functional status was examined with linear mixed-effect regression analyses based on two-level random-intercept models. The data points represent the mean global cognition z-scores in panel (**A**) and mean CDR-SB scores in panel (**B**), while the whiskers represent the standard error, according to each RHR-group. CDR-SB, clinical dementia rating—sum of boxes; RHR, resting heart rate.

Over a median follow-up of 60 (IQR 36–60) months, longitudinal data were available in 604 subjects. Compared to RHR 60–69 bpm, RHR ≥70 bpm demonstrated an accelerated decline in global cognition (*P*_interaction_ = 0.035, Table 2) on multivariable adjustment for age, sex, education, ApoE4 status, SBP, history of hypertension, diabetes, rate-limiting medications, baseline MMSE and cognitive enhancers. Bradycardia trended towards accelerated decline in global cognition when compared to RHR 60–69 bpm (*P*_interaction_ = 0.071), but the rate of decline was similar between bradycardia and elevated RHR (*P*_interaction_ = 1.000) on multivariable adjustment. Conversely, bradycardia was independently associated with accelerated functional decline (*P*_interaction_ = 0.016, Table 2) when compared to RHR 60–69 bpm. The rate of decline in global cognition and functional status are shown in Fig. 2. With respect to individual cognitive domains, attention was the only cognitive domain associated with an accelerated decline with RHR ≥70 bpm after correction for multiple testing (*P*_interaction_ = 0.002). The rate of decline in global cognition and functional deficits did not differ between bradycardia and RHR ≥70 bpm.

**Table 2:** Longitudinal association of RHR with rate of cognitive and functional decline

	Model A			Model B		
	ß*	95% CI	*P*	B*	95% CI	*P*
Global cognition						
RHR <60 bpm versus 60–69 bpm*time	−0.06	−0.12, 0.01	0.072	−0.06	−0.12, 0.005	0.071
RHR ≥70 bpm versus 60–69 bpm*time	−0.06	−0.12, −0.01	0.025	−0.06	−0.11, −0.004	0.035
RHR ≥70 bpm versus <60 bpm*time	−0.004	−0.07, 0.06	0.892	−7.56 × 10^−6^	−0.06, 0.06	1.000
CDR-SB						
RHR <60 bpm versus 60–69 bpm*time	0.15	0.03, 0.26	0.011	0.14	0.03, 0.26	0.016
RHR ≥70 bpm versus 60–69 bpm*time	0.06	−0.04, 0.15	0.275	0.04	−0.06, 0.14	0.413
RHR ≥70 bpm versus <60 bpm*time	−0.09	−0.21, 0.02	0.108	−0.10	−0.22, 0.02	0.096
Memory						
RHR <60 bpm versus 60–69 bpm*time	−0.04	−0.07, −0.002	0.041	−0.04	−0.07, −0.003	0.035
RHR ≥70 bpm versus 60–69 bpm*time	−0.03	−0.06, −0.001	0.043	−0.03	−0.06, −0.0001	0.049
RHR ≥70 bpm versus <60 bpm*time	0.005	−0.03, 0.04	0.762	0.01	−0.03, 0.04	0.679
Visuoconstruction						
RHR <60 bpm versus 60–69 bpm*time	−0.03	−0.09, 0.03	0.317	−0.03	−0.09, 0.03	0.287
RHR ≥70 bpm versus 60–69 bpm*time	−0.04	−0.09, 0.01	0.083	−0.04	−0.09, 0.01	0.108
RHR ≥70 bpm versus <60 bpm*time	−0.01	−0.07, 0.04	0.621	−0.01	−0.07, 0.05	0.752
Visuomotor						
RHR <60 bpm versus 60–69 bpm*time	−0.01	−0.03, 0.01	0.404	−0.01	−0.03, 0.01	0.482
RHR ≥70 bpm versus 60–69 bpm*time	−0.02	−0.04, −0.005	0.015	−0.02	−0.04, −0.003	0.024
RHR ≥70 bpm versus <60 bpm*time	−0.01	−0.04, 0.01	0.203	−0.01	−0.04, 0.01	0.218
Language						
RHR <60 bpm versus 60–69 bpm*time	−0.12	−0.29, 0.05	0.164	−0.12	−0.29, 0.05	0.169
RHR ≥70 bpm versus 60–69 bpm*time	−0.09	−0.24, 0.05	0.210	−0.09	−0.23, 0.06	0.249
RHR ≥70 bpm versus <60 bpm*time	0.03	−0.14, 0.20	0.752	0.03	−0.15, 0.22	0.713
Attention						
RHR <60 bpm versus 60–69 bpm*time	−0.03	−0.07, 0.01	0.178	−0.03	−0.07, 0.02	0.206
RHR ≥70 bpm versus 60–69 bpm*time	−0.06	−0.10, −0.02	0.002	−0.06	−0.10, −0.02	0.002
RHR ≥70 bpm versus <60 bpm*time	−0.03	−0.07, 0.01	0.168	−0.03	−0.07, 0.01	0.161
Executive function						
RHR <60 bpm versus 60–69 bpm*time	−0.02	−0.07, 0.02	0.320	−0.02	−0.07, 0.02	0.320
RHR ≥70 bpm versus 60–69 bpm*time	−0.02	−0.06, 0.03	0.455	−0.01	−0.05, 0.03	0.532
RHR ≥70 bpm versus <60 bpm*time	0.01	−0.04, 0.06	0.724	0.01	−0.04, 0.06	0.652

Model A: adjusted for age, sex, education.

Model B: adjusted for age, sex, education, ApoE4 status, SBP, hypertension, diabetes mellitus, rate-limiting medications, baseline MMSE, cognitive enhancers.

ApoE4, apolipoprotein E4; BMI, body mass index; CDR-SB, clinical dementia rating—sum of boxes; MMSE, mini mental state examination; RHR, resting heart rate; SBP, systolic blood pressure.

### Association of RHR with neuroimaging markers

Among neuroimaging parameters studied on baseline brain MRI (Table 3), bradycardia was associated only with significantly lower grey matter volume compared to RHR 60–69 bpm after multivariable adjustment for age, sex, BMI, hypertension and diabetes (*P* < 0.05). By contrast, elevated RHR was associated with 2-times increased burden of cortical infarcts, 1.8-times increased burden of CMB and 1.8-times increased burden of lacunes, when compared to RHR 60–69 bpm, after multivariable adjustment for clinical factors (*P* < 0.05, Table 3). When compared to bradycardia, elevated RHR was associated with higher WMH ratio and 2-times higher burden of CMB (*P* < 0.05).

**Table 3:** Cross-sectional association of RHR with neuroimaging markers of cerebrovascular disease

	Model A			Model B		
	ß	95% CI	*P*	ß	95% CI	*P*
GMV ratio						
RHR <60 bpm versus 60–69 bpm	−0.57	−1.08, −0.05	0.032	−0.55	−1.06, −0.03	0.037
RHR ≥70 bpm versus 60–69 bpm	−0.36	−0.80, 0.09	0.118	−0.30	−0.74, 0.15	0.187
RHR ≥70 bpm versus <60 bpm	0.21	−0.29, 0.71	0.412	0.25	−0.25, 0.75	0.330
WMV ratio						
RHR <60 bpm versus 60–69 bpm	−0.13	−0.65, 0.39	0.617	−0.12	−0.63, 0.40	0.659
RHR ≥70 bpm versus 60–69 bpm	0.07	−0.38, 0.51	0.768	0.13	−0.31, 0.58	0.559
RHR ≥70 bpm versus <60 bpm	0.20	−0.31, 0.71	0.447	0.29	−0.22, 0.79	0.268
HV ratio						
RHR <60 bpm versus 60–69 bpm	−0.005	−0.02, 0.01	0.515	−0.004	−0.02, 0.01	0.589
RHR ≥70 bpm versus 60–69 bpm	−0.003	−0.02, 0.01	0.601	−0.003	−0.02, 0.01	0.681
RHR ≥70 bpm versus <60 bpm	0.002	−0.01, 0.02	0.837	0.001	−0.01, 0.02	0.851
WMH ratio						
RHR <60 bpm versus 60–69 bpm	−1.87	−5.29, 1.56	0.285	−1.88	−5.27, 1.51	0.277
RHR ≥70 bpm versus 60–69 bpm	1.67	−1.27, 4.61	0.265	1.57	−1.35, 4.49	0.290
RHR ≥70 bpm versus <60 bpm	3.53	0.22, 6.85	0.037	3.59	2.30, 6.89	0.033

	IRR	95%CI	*P*	ARR	95%CI	*P*
Cortical infarcts						
RHR <60 bpm versus 60–69 bpm	1.80	0.97–3.32	0.061	1.83	0.98–3.42	0.056
RHR ≥70 bpm versus 60–69 bpm	1.89	1.09–3.27	0.022	1.96	1.12–3.41	0.018
RHR ≥70 bpm versus <60 bpm	1.04	0.62–1.75	0.885	1.04	0.62–1.75	0.885
CMB						
RHR <60 bpm versus 60–69 bpm	0.84	0.65–1.10	0.207	0.91	0.69–1.19	0.468
RHR ≥70 bpm versus 60–69 bpm	1.94	1.56–2.41	<0.001	1.80	1.44–2.24	<0.001
RHR ≥70 bpm versus <60 bpm	2.23	1.73–2.87	<0.001	2.23	1.73–2.89	<0.001
Lacunes						
RHR <60 bpm versus 60–69 bpm	1.25	0.86–1.81	0.242	1.25	0.85–1.83	0.255
RHR ≥70 bpm versus 60–69 bpm	1.76	1.28–2.42	<0.001	1.78	1.29–2.47	<0.001
RHR ≥70 bpm versus <60 bpm	1.37	0.98–1.93	0.065	1.37	0.98–1.93	0.065

Model A: adjusted for age, sex, BMI.

Model B: adjusted for age, sex, BMI, hypertension, diabetes mellitus.

ARR, adjusted rate ratio; BMI, body mass index; CMB, cerebral microbleeds; GMV, grey matter volume; HV, hippocampal volume; IRR, incidence rate ratio; RHR, resting heart rate; WMH, white matter hyperintensity.

### Association of RHR with circulating biomarkers

Baseline concentrations of pTau-181 were higher among subjects with bradycardia compared to both RHR 60–69 bpm and RHR ≥70 bpm (*P* < 0.05), while baseline concentrations of NFL were significantly higher among subjects with bradycardia compared to RHR 60–69 bpm only (*P* < 0.05, Table 1). Circulating levels of NFL or pTau-181 were similar between RHR 60–69 bpm and ≥70 bpm (*P* > 0.05), and GFAP levels between all 3 RHR groups (*P* = 0.981, Table 1).

The associations of RHR with circulating biomarker concentrations (log-transformed) are shown in Table 4. In robust regression analyses, only bradycardia was associated with significantly higher levels of NFL and pTau-181 after adjustment for age, sex, BMI, hypertension, diabetes and cognitive enhancers when compared to RHR 60–69 bpm (*P* < 0.05). Similarly, bradycardia was associated with higher levels of pTau-181 when compared to RHR ≥70 bpm in multivariable analyses (*P* < 0.001). There were no associations between RHR and circulating GFAP levels (*P* > 0.05, Table 4).

**Table 4:** Cross-sectional association of RHR with circulating biomarkers (log-transformed)

	Model A			Model B		
	ß	95% CI	*P*	ß*	95% CI	*P*
pTau-181						
RHR <60 bpm versus 60–69 bpm	0.09	0.04, 0.14	0.001	0.09	0.03, 0.14	0.001
RHR ≥70 bpm versus 60–69 bpm	0.002	−0.04, 0.05	0.920	−0.01	−0.05, 0.04	0.742
RHR ≥70 bpm versus <60 bpm	−0.09	−0.14, −0.04	0.001	−0.09	−0.14, −0.04	<0.001
NFL						
RHR <60 bpm versus 60–69 bpm	0.06	0.01, 0.11	0.021	0.05	0.001, 0.10	0.046
RHR ≥70 bpm versus 60–69 bpm	0.03	−0.01, 0.08	0.137	0.02	−0.02, 0.06	0.321
RHR ≥70 bpm versus <60 bpm	−0.02	−0.07, 0.02	0.328	−0.02	−0.07, 0.02	0.300
GFAP						
RHR <60 bpm versus 60–69 bpm	−0.01	−0.18, 0.16	0.905	−0.02	−0.18, 0.14	0.785
RHR ≥70 bpm versus 60–69 bpm	0.01	−0.14, 0.15	0.906	0.001	−0.14, 0.14	0.991
RHR ≥70 bpm versus <60 bpm	0.02	−0.14, 0.19	0.800	0.04	−0.12, 0.20	0.646
pTau-217						
RHR <60 bpm versus 60–69 bpm	0.02	0.002, 0.04	0.028	0.02	0.001, 0.04	0.036
RHR ≥70 bpm versus 60–69 bpm	−0.01	−0.02, 0.01	0.346	−0.01	−0.03, 0.004	0.149
RHR ≥70 bpm versus <60 bpm	−0.03	−0.05, −0.01	0.002	−0.03	−0.05, −0.01	0.001

Model A: adjusted for age, sex and BMI.

Model B: adjusted for age, sex, BMI, hypertension, diabetes mellitus, cognitive enhancers.

BMI, body mass index; NFL, neurofilament light chain; RHR, resting heart rate.

Circulating pTau-217 protein levels were significantly higher among subjects with bradycardia (11.6 [inter-quartile range 10.9–12.4] NPQ) compared to RHR 60–69 bpm (11.2 [IQR 10.7–12.1] NPQ) (*P* = 0.043) and elevated RHR (11.2 [IQE 10.7–11.9] NPQ) (*P* = 0.001), but were similar between RHR 60–69 bpm and elevated RHR (*P* = 0.262). In robust regression analyses, bradycardia was associated with significantly higher pTau-217 levels when adjusted for age, sex, BMI, hypertension, diabetes and cognitive enhancers compared to RHR 60–69 bpm (*P* = 0.036) and elevated RHR (*P* = 0.001) (Table 4).

### Sensitivity analyses

In sensitivity analyses including only subjects without pre-existing dementia (*n* = 390, mean age 71.4 ± 7.9 years, 54% female, mean education 8.2 ± 5.2 years, 33% NCI, 67% CIND), 144 (37%) had RHR 60–69 bpm, 88 (23%) had bradycardia and 158 (41%) had RHR ≥70 bpm. The association of RHR ≥70 bpm with accelerated global cognitive decline (*P*_interaction_ = 0.030), and bradycardia with accelerated functional decline (*P*_interaction_ = 0.018) held true on multivariable adjustment (Supplementary Table 4). In addition, subjects with bradycardia also demonstrated an accelerated decline in global cognition when compared to RHR 60–69 bpm (*P*_interaction_ = 0.039) on multivariable adjustment for age, sex, education, ApoE4 status, SBP, hypertension, diabetes, rate-limiting mediations, baseline MMSE and cognitive enhancers. With respect to individual cognitive domains, the independent association between bradycardia (adjusted for age, sex, education) and accelerated decline in memory compared to RHR 60–69 bpm was attenuated after adjusting additionally for clinical morbidities, medications and baseline MMSE (*P*_interaction_ correction for multiple testing > 0.0083).

With regard to neuroimaging and circulating biomarkers, only elevated RHR ≥70 bpm remained significantly associated with a higher burden of cortical infarcts and lacunes, and higher WMH ratio when compared to RHR 60–69 bpm (*P* < 0.05, Supplementary Table 5). The significant associations of bradycardia with higher pTau-181 levels persisted when compared to both RHR 60–69 and ≥70 bpm (Supplementary Table 6, *P* < 0.05), but its association with circulating NFL was attenuated.

## Discussion

In this prospective study of memory clinic subjects with comprehensive cognitive phenotyping, neuroimaging and circulating biomarkers, we present novel findings of a U-shaped association between RHR and cognitive decline, with distinct associations between bradycardia and elevated RHR with neuroimaging and circulating biomarkers. Longitudinally, decline in global cognition was accelerated when RHR was ≥70 bpm and in the subset of bradycardia subjects without dementia, while decline in functional impairment was accelerated with bradycardia. The mechanisms underlying RHR-related cognitive dysfunction appear distinct between RHR groups, evidenced by (i) the associations between RHR ≥70 bpm and increased CeVD burden including cortical infarcts, CMB and lacunes, and worse executive function suggesting a vascular-mediated pathology related to cerebral vascular small disease (CSVD); (ii) associations between bradycardia and lower grey matter volume and higher levels of circulating pTau-181, pTau-217 and NFL, suggesting neurodegenerative pathology. Our findings extend current knowledge in highlighting novel associations of RHR with worse cognitive and functional decline, and the discrete mechanisms underpinning these associations.

### Association of RHR ≥70 bpm with cognitive decline

When compared to RHR 60–69 bpm, RHR ≥70 bpm was associated with accelerated cognitive decline, based on reductions in MMSE scores, in the Swedish National Study on Aging and Care in Kungsholmen (SNAC-K), the Chinese Longitudinal Healthy Longevity Survey (CLHLS) and Prevention Regimen for Effectively Avoiding Second Stroke (PROFESS) trial of ischaemic patients.^4-6^ Similarly, the Atherosclerosis Risk in Communities (ARIC) study, which used a composite score derived from Delayed Word Recall, Digit Symbol Substitution and word fluency, reported greater global cognitive decline over 20 years with RHR ≥70 bpm, in comparison with RHR <60 bpm.^8^ By contrast, the Women’s Health Initiative Memory Study (WHIMS) did not find associations between RHR and cognitive impairment in post-menopausal women without cardiovascular disease.^9^ In concert with most studies, we report consistent associations of higher RHR (≥70 bpm) with worse cognitive function cross-sectionally and accelerated decline in global cognition longitudinally, when compared to a RHR of 60–69 bpm.

Elevated RHR has been posited to affect cognition through shared cardiovascular risk factors, sympathetic hyperactivity, increased pulse pressure and arterial stiffness, subclinical inflammation, endothelial dysfunction and atherosclerosis.^5,21,22^ Data on RHR and neuroimaging findings are scarce, but WHIMS reported an association between elevated RHR with greater ischaemic lesion volumes, and grey and white matter volumes, and increased ischaemic lesion volumes longitudinally.^9^ Others have shown associations between higher nocturnal heart rates and WMH burden,^23^ and higher RHR with increased arterial stiffness,^24^ a marker of vascular cognitive impairment mediated in part by CSVD.^25^ We extend current literature by demonstrating in these subjects, a greater burden of cortical infarcts and a wider array of CSVD features (lacunes, CMB). The accelerated decline in global cognition with higher RHR is likely mediated by an underlying vascular-mechanism,^26^ supported further by its association with impaired executive function, a marker of vascular cognitive impairment,^26^ and lack of association with pTau-181, a marker of cerebral amyloid deposition.^27^ Additionally, higher RHR may also reflect reduced physical activity and cardiovascular fitness, increasing the risk of cognitive impairment and decline.^28^

### Association of bradycardia with cognitive decline

The Multidomain Interventions to Delay Dementia and Disability in Rural China (MIND-China) study demonstrated cross-sectional associations of lower RHR with lower MMSE and verbal fluency scores.^7^ Improvements in cognitive function and cerebral perfusion after pacemaker implantation further support the relevance of bradycardia to worse cognition.^10,11^ However, unlike higher RHR, the association of bradycardia with cognitive decline is less evident. In SNAC-K, CLHLS and ARIC^5,6,8^ there were no associations between bradycardia (<60 bpm) and incident dementia or cognitive decline when compared to RHR 60–69 bpm. A preliminary J-shaped association between RHR and incident dementia in SNAC-K, suggesting higher risk with RHR <50 bpm, was no longer apparent following comprehensive adjustment for risk factors.^5^

We report novel associations of bradycardia with greater cross-sectional cognitive impairment and accelerated functional decline longitudinally and, in a subset of pre-dementia subjects, accelerated decline in global cognition, when compared to those with a RHR of 60–69 bpm. Compared to earlier reports, we employed comprehensive neuropsychological assessments which afford greater sensitivity in detecting cognitive decline than MMSE.^29^ Notably, education levels were similar between RHR-subgroups in our study whereas in earlier cohorts, subjects with bradycardia had higher education vis-à-vis other RHR groups.^5,7,8^ Subjects in ARIC were also younger, with a lower prevalence of cardiovascular disease,^8^ and therefore a different dementia-risk profile compared to our cohort.

Interestingly, the associations of bradycardia with neuroimaging and circulating biomarkers were distinct from higher RHR. Compared to RHR 60–69 bpm, bradycardia was associated with reduced GMV, and higher circulating pTau-181, pTau-217 and NFL levels, suggesting a neurodegenerative pathology. Grey matter atrophy has been associated with cerebral hypoperfusion in Alzheimer’s disease,^30,31^ while NFL, elevated in axonal damage, is a recognized marker of the amyloid-beta-tau-neurodegeneration classification in Alzheimer’s disease.^32^ In addition, bradycardia displayed a striking association with higher levels of pTau-181 and pTau-217, robust markers of cerebral tau and Aß pathologies.^33^ This association with neurodegeneration could be explained by several mechanisms. First, bradycardia may lead to impaired cerebral perfusion and disrupted cerebral autoregulation from chronic hemodynamic perturbations,^34,35^ resulting in impaired cerebral clearance of Aß. Second, deposition of amyloid fibres from circulating Aß, due to a common systemic disease involving both the heart and brain, or a primary neurological disease affecting the heart, has also been described. Indeed, intramyocardial Aß deposits have been discovered in patients with Alzheimer’s disease and diastolic dysfunction.^13^ Third, subtypes of neurodegeneration may manifest bradycardia and cognitive decline unrelated to CSVD, including the relatively common dementia with Lewy bodies which is associated with sick sinus syndrome,^36^ and frontoparietal dementia with autonomic dysfunction.^37^ Finally, cognitive enhancers commonly prescribed in Alzheimer’s disease may be associated with bradycardia. However, a network analysis of five randomized trials involving 1290 subjects did not find significant differences in bradycardia events with these medications.^38^ The observed associations of bradycardia with accelerated cognitive decline and pTau-181 in our study were independent of cognitive enhancers, suggesting that these medications do not mediate the relationship.

### Clinical implications

The extent of cognitive impairment and rate of cognitive decline was similar among those with bradycardia and higher RHR (together including 65% of our patient population), supporting a U-shaped association between RHR and cognitive impairment/decline. While earlier studies have emphasized the prognostic significance of elevated RHR, our findings indicate that bradycardia (23% of our cohort) also portends worse cognitive trajectories and functional decline. Our findings also suggest distinct mechanisms underlying this U-shaped association, and invite further study into the underlying pathophysiological mechanisms.

### Limitations

Compared to earlier studies,^4-9^ our sample size was smaller and comprised older Asian subjects from memory clinics with a higher prevalence of cardiovascular disease. Findings from this study may therefore not be generalizable to younger patients at lower risk of cognitive decline or Western populations. The small sample size also precludes ethnic comparisons in the association of RHR and cognitive decline. RHR measurements were from a single time point and serial changes in RHR and cognitive decline were not examined. While we recognize the protective effect of physical activity and exercise on cognition,^28^ these data were not available. The associations of RHR with neuroimaging and circulating biomarkers were cross-sectional in nature and do not allow the assessment of causality of cognitive decline. Finally, circulating pTau-217 levels were obtained from proteomic assays and therefore qualitative rather than quantitative.

## Conclusion

Compared to RHR 60–69 bpm, bradycardia and elevated RHR ≥70 bpm (together over 70% of our study population) were associated with worse cognitive trajectories and may be underpinned by distinct mechanisms. Further elucidation of the mechanisms linking RHR, in particular bradycardia, with cognitive decline is needed.

## Supplementary Material

fcaf413_Supplementary_Data

## Data Availability

The data that support the findings of this study are available from the corresponding author upon reasonable request.
