# Supplementary material for: U-shaped association of resting heart rate with cognitive decline
Source: Brain Commun. 2025 Oct 21;7(6):fcaf413. doi: 10.1093/braincomms/fcaf413 (PMC12596192; doi:10.1093/braincomms/fcaf413)
Supplement: fcaf413_Supplementary_Data [file fcaf413_supplementary_data.pdf]

Supplementary Table 1. Neuropsychological Assessment

Domain	Test
Global Dementia Screening	Mini Mental State Examination (MMSE)<br>Montreal Cognitive Assessment (MocA)
Attention	Digit Span Forward<br>Digit Span Backward
Executive Function	Color Trails Test 1<br>Color Trails Test 2<br>Animal Naming (Fluency)
Language	Modified Boston Naming test
Visuomotor Speed	Symbol Digit Modalities test
Visuoconstruction	Rey Complex Figure test Copy
Memory	Rey Complex Figure test – Immediate recall (3 minutes)<br>Rey Complex Figure test – Delayed recall (30 minutes)<br>Rey Complex Figure test – Recognition<br>Hopkins Verbal Learning test – Immediate<br>Hopkins Verbal Learning test – Delayed<br>Hopkins Verbal Learning test – Recognition<br>Recognition Discrimination Index

Supplementary Table 2. Brain magnetic resonance imaging sequences

T1-weighted MRI	High-resolution T1-weighted Magnetization Prepared Rapid Gradient Recalled Echo (MPRAGE) or similar versions were acquired for the whole brain or specific regions of interest.
T2-weighted MRI	T2-weighted images were also acquired using a double spin echo.
Fluid Attenuated Inversion Recovery (FLAIR)	FLAIR data were acquired using FSE- IR sequence.
Diffusion MR Imaging (dMRI)	Diffusion MRI data were acquired using a single-shell and multi-shell multiband echo-planar imaging (EPI) sequence. Additional several images without diffusion weighting (b-value=0 sec/mm2) were acquired.
Blood Oxygen Level Dependent (BOLD) functional MRI	BOLD functional MRI was acquired with subjects at rest. Multi-slice echo-planar imaging (EPI) (single or multiband) were used to cover the whole brain. Concurrent autonomic tracking was used such as eye-tracking.
Susceptibility weighted imaging (SWI)	SWI data were acquired during GRE sequence.
Arterial Spin Labelling (ASL)	ASL processing was performed with the ExploreASL software based on SPM and Matlab (MathWorks, MA). Image processing includes motion correction and rigid-body registration of the CBF map to a gray matter map and normalized into a common space using Diffeomorphic Anatomical Registration analysis using Exponentiated Lie

	algebra (DARTEL). From the CBF map, two ASL parameters were acquired: CBF and sCoV. CBF reflects perfusion in mL blood/100g tissue/minute and were calculated in the gray and white matter ROIs. sCoV, which was shown to serve as a proxy of ATT, were defined as the standard deviation (SD) of the CBF/mean CBF within the region of interest.
Time of flight Magnetic Resonance Angiography (MRA)	High-resolution 3D TOF MRA was acquired using a 3D fast low-angle shot sequence (gradient recalled echo) with an isotropic voxel size.
Quality Assurance (QA)	Quality assurance scans were performed on the day when the patient scan is scheduled. The QA scan is acquired using a gradient echo pulse sequence. The QA images were analyzed in regard to signal to noise ratio (SNR), spatial resolution, slice thickness and location.
Subjects underwent multimodal MRI brain scan at baseline and again at two years. MRI scans were acquired at the Clinical Imaging Research Centre, on a 3-Tesla whole-body MRI scanner with a state-of-the-art 32-channel head coil. Functional and anatomical image acquisition were undertaken with the following protocol and sequences.	

Supplementary Table 3. Cross-sectional association of RHR with cognitive function

	Model A			Model B		
	ß	95%CI	p	ß	95%CI	p
Global cognition						
RHR <60bpm vs 60-69bpm	-0.62	-1.08, -0.165	0.009	-0.56	-1.03, -0.09	0.020
RHR ≥70bpm vs 60-69bpm	-0.51	-0.91, -0.12	0.011	-0.51	-0.92, -0.11	0.013
RHR ≥70bpm vs <60bpm	0.10	-0.34, 0.55	0.655	0.02	-0.47, 0.51	0.938
CDR-SB						
RHR <60bpm vs 60-69bpm	0.79	0.01, 1.58	0.048	0.73	-0.06, 1.53	0.071
RHR ≥70bpm vs 60-69bpm	0.91	0.25, 1.59	0.008	0.91	0.22, 1.60	0.010
RHR ≥70bpm vs <60bpm	0.12	-0.64, 0.88	0.756	0.24	-0.61, 1.09	0.578
Memory						
RHR <60bpm vs 60-69bpm	-0.12	-0.41, 0.17	0.410	-0.06	-0.35, 0.22	0.664
RHR ≥70bpm vs 60-69bpm	-0.15	-0.40, 0.09	0.216	-0.16	-0.41, 0.09	0.204
RHR ≥70bpm vs <60bpm	-0.03	-0.31, 0.24	0.810	-0.09	-0.39, 0.21	0.551

Visuoconstruction						
RHR <60bpm vs 60-69bpm	-0.42	-0.79, -0.04	0.031	-0.38	-0.77, -0.001	0.049
RHR ≥70bpm vs 60-69bpm	-0.38	-0.70, -0.06	0.020	-0.38	-0.71, -0.05	0.025
RHR ≥70bpm vs <60bpm	0.03	-0.33, 0.40	0.856	0.01	-0.39, 0.41	0.959
Visuomotor speed						
RHR <60bpm vs 60-69bpm	-0.20	-0.40, 0.003	0.053	-0.17	-0.37, 0.03	0.096
RHR ≥70bpm vs 60-69bpm	-0.15	-0.32, -0.02	0.082	-0.16	-0.33, 0.02	0.079
RHR ≥70bpm vs <60bpm	0.05	-0.15, 0.24	0.639	0.01	-0.20, 0.23	0.895
Language						
RHR <60bpm vs 60-69bpm	-1.03	-1.94, -0.12	0.027	-1.02	-1.95, -0.08	0.033
RHR ≥70bpm vs 60-69bpm	-0.68	-1.46, 0.10	0.088	-0.64	-1.44, 0.16	0.117
RHR ≥70bpm vs <60bpm	0.35	-0.53, 1.23	0.431	0.24	-0.73, 1.22	0.622
Attention						
RHR <60bpm vs 60-69bpm	-0.32	-0.57, -0.08	0.010	-0.31	-0.56, -0.07	0.013
RHR ≥70bpm vs 60-69bpm	-0.25	-0.45, -0.04	0.022	-0.26	-0.48, -0.05	0.016
RHR ≥70bpm vs <60bpm	0.08	-0.16, 0.31	0.518	0.06	-0.18, 0.31	0.608

Executive function						
RHR <60bpm vs 60-69bpm	-0.54	-0.94, -0.12	0.012	-0.43	-0.85, -0.02	0.040
RHR ≥70bpm vs 60-69bpm	-0.58	-0.93, -0.23	0.001	-0.59	-0.95 -0.24	0.001
RHR ≥70bpm vs <60bpm	-0.05	-0.44, 0.35	0.820	-0.16	-0.59, 0.27	0.462

Model A: adjusted for age, sex, education

Model B: adjusted for age, sex, education, ApoE4 status, SBP, hypertension, diabetes mellitus, rate-limiting medications

AF, atrial fibrillation; ApoE4, apolipoprotein E4; BMI, body mass index; CDR-SB, Clinical Dementia Rating – Sum of Boxes; IHD, ischemic heart disease; MMSE, mini mental state exam; MOCA, Montreal Cognitive Assessment; NPS, neuropsychiatric symptom; RHR, resting heart rate; SBP, systolic blood pressure

Supplementary Table 4. Association of RHR with longitudinal decline in subjects without preexisting dementia

	Model A			Model B		
	ß*	95%CI	p	ß*	95%CI	p
Global cognition						
RHR <60bpm vs 60-69bpm*time	-0.06	-0.12, -0.01	0.023	-0.06	-0.12, -0.01	0.030
RHR ≥70bpm vs 60-69bpm*time	-0.05	-0.10, -0.004	0.033	-0.05	-0.10, -0.003	0.039
RHR ≥70bpm vs <60bpm*time	0.01	-0.04, 0.07	0.671	0.01	-0.05, 0.07	0.734
CDR-SB						
RHR <60bpm vs 60-69bpm*time	0.15	0.03, 0.26	0.012	0.14	0.03, 0.25	0.018
RHR ≥70bpm vs 60-69bpm*time	0.06	-0.04, 0.15	0.268	0.05	-0.05, 0.15	0.300
RHR ≥70bpm vs <60bpm*time	-0.09	-0.20, 0.02	0.124	-0.09	-0.21, 0.04	0.177
Memory						
RHR <60bpm vs 60-69bpm*time	-0.06	-0.10, -0.02	0.008	-0.06	-0.10, -0.02	0.009
RHR ≥70bpm vs 60-69bpm*time	-0.04	-0.08, -0.004	0.031	-0.04	-0.08, -0.004	0.032
RHR ≥70bpm vs <60bpm*time	0.02	-0.03, 0.06	0.450	0.02	-0.03, 0.06	0.455

Visuoconstruction						
RHR <60bpm vs 60-69bpm*time	-0.06	-0.12, 0.004	0.067	-0.06	-0.12, 0.01	0.071
RHR ≥70bpm vs 60-69bpm*time	-0.06	-0.11, -0.003	0.040	-0.06	-0.11, -0.003	0.040
RHR ≥70bpm vs <60bpm*time	0.002	-0.06, 0.07	0.963	0.001	-0.07, 0.07	0.985
Visuomotor						
RHR <60bpm vs 60-69bpm*time	-0.02	-0.04, 0.01	0.276	-0.01	-0.04, 0.02	0.369
RHR ≥70bpm vs 60-69bpm*time	-0.02	-0.05, -0.004	0.095	-0.02	-0.04, 0.004	0.108
RHR ≥70bpm vs <60bpm*time	-0.01	-0.03, 0.02	0.725	-0.01	-0.04, 0.02	0.625
Language						
RHR <60bpm vs 60-69bpm*time	-0.06	-0.18, 0.06	0.352	-0.06	-0.18, 0.07	0.380
RHR ≥70bpm vs 60-69bpm*time	-0.03	-0.14, 0.07	0.543	-0.03	-0.14, 0.08	0.596
RHR ≥70bpm vs <60bpm*time	0.03	-0.10, 0.15	0.689	0.03	-0.11, 0.16	0.706
Attention						
RHR <60bpm vs 60-69bpm*time	-0.04	-0.09, 0.001	0.057	-0.04	-0.09, 0.01	0.089
RHR ≥70bpm vs 60-69bpm*time	-0.04	-0.08, -0.02	0.042	-0.04	-0.08, 0.001	0.054
RHR ≥70bpm vs <60bpm*time	0.003	-0.04, 0.05	0.890	0.001	-0.05, 0.05	0.978

Executive function						
RHR <60bpm vs 60-69bpm*time	-0.04	-0.10, 0.01	0.129	-0.04	-0.09, 0.02	0.158
RHR ≥70bpm vs 60-69bpm*time	-0.03	-0.08, 0.02	0.217	-0.03	-0.08, 0.02	0.212
RHR ≥70bpm vs <60bpm*time	0.13	-0.04, 0.07	0.657	0.01	-0.05, 0.07	0.754

Model A: adjusted for age, sex, education

Model B: adjusted for age, sex, education, ApoE4 status, SBP, hypertension, diabetes mellitus, rate-limiting medications, baseline MMSE; cognitive enhancers

ApoE4, apolipoprotein E4; BMI, body mass index; CDR-SB, Clinical Dementia Rating – Sum of Boxes; RHR, resting heart rate; SBP, systolic blood pressure

Supplementary Table 5. Cross-sectional association of RHR with neuroimaging markers of cerebrovascular disease in subjects without preexisting dementia

	Model A			Model B		
	ß	95%CI	p value	ß	95%CI	p value
GMV ratio						
RHR <60bpm vs 60-69bpm	-0.27	-0.82, 0.28	0.332	-0.25	-0.80, 0.29	0.364
RHR ≥70bpm vs 60-69bpm	-0.26	-0.73, 0.21	0.272	-0.27	-0.75, 0.20	0.257
RHR ≥70bpm vs <60bpm	0.01	-0.54, 0.55	0.978	-0.02	-0.57, 0.53	0.942
WMV ratio						
RHR <60bpm vs 60-69bpm	-0.19	-0.78, 0.41	0.537	-0.14	-0.73, 0.45	0.643
RHR ≥70bpm vs 60-69bpm	0.10	-0.41, 0.61	0.709	0.22	-0.29, 0.73	0.400
RHR ≥70bpm vs <60bpm	0.27	-0.32, 0.87	0.365	0.38	-0.21, 0.98	0.208
HV ratio						
RHR <60bpm vs 60-69bpm	-0.01	-0.02, 0.01	0.318	-0.01	-0.02, 0.01	0.401
RHR ≥70bpm vs 60-69bpm	-0.005	-0.02, 0.01	0.505	-0.004	-0.02, 0.01	0.530

RHR ≥70bpm vs <60bpm	0.003	-0.01, 0.02	0.667	0.002	-0.01, 0.02	0.767
WMH ratio						
RHR <60bpm vs 60-69bpm	-0.14	-0.36, 0.08	0.212	-0.15	-0.37, 0.07	0.185
RHR ≥70bpm vs 60-69bpm	0.08	-0.11, 0.27	0.433	0.07	-0.12, 0.26	0.464
RHR ≥70bpm vs <60bpm	0.22	-0.003, 0.44	0.053	0.22	0.001, 0.45	0.049
	IRR	95%CI	p value	ARR	95%CI	p value
Cortical infarcts						
RHR <60bpm vs 60-69bpm	3.16	0.91-10.96	0.070	3.81	1.06-13.64	0.040
RHR ≥70bpm vs 60-69bpm	4.97	1.65-14.93	0.004	5.53	1.77-17.24	0.003
RHR ≥70bpm vs <60bpm	1.55	0.65-3.69	0.321	1.41	0.57-3.48	0.451
CMB						
RHR <60bpm vs 60-69bpm	1.17	0.82-1.54	0.387	1.28	0.90-1.83	0.174
RHR ≥70bpm vs 60-69bpm	1.08	0.79-1.47	0.646	1.18	0.86-1.61	0.316
RHR ≥70bpm vs <60bpm	0.84	0.59-1.20	0.346	0.87	0.61-1.25	0.453
Lacunes						
RHR <60bpm vs 60-69bpm	1.23	0.72-2.11	0.442	1.23	0.71-2.12	0.459

RHR ≥70bpm vs 60-69bpm	1.78	1.14-2.81	0.012	1.69	1.07-2.69	0.025
RHR ≥70bpm vs <60bpm	1.44	0.87-2.37	0.153	1.38	0.83-2.29	0.215

Model A: adjusted for age, sex, BMI

Model B: adjusted for age, sex, BMI, hypertension, diabetes mellitus

ARR, adjusted rate ratio; BMI, body mass index; CMB, cerebral microbleeds; GMV, grey matter volume; HV, hippocampal volume; IRR, incidence rate ratio; RHR, resting heart rate

Supplementary Table 6. Cross-sectional association of RHR with circulating biomarkers (log-transformed) in subjects without preexisting dementia

	Model A			Model B		
	ß	95%CI	p	ß*	95%CI	p
pTau-181						
RHR <60bpm vs 60-69bpm	0.10	0.04-0.15	0.001	0.09	0.03-0.15	0.002
RHR ≥70bpm vs 60-69bpm	0.03	-0.02, 0.08	0.175	0.03	-0.02, 0.08	0.208
RHR ≥70bpm vs <60bpm	-0.06	-0.12, -0.01	0.024	-0.06	-0.12, -0.01	0.028
NFL						
RHR <60bpm vs 60-69bpm	0.05	-0.004, 0.10	0.068	0.03	-0.02, 0.09	0.182
RHR ≥70bpm vs 60-69bpm	0.03	-0.01, 0.08	0.147	0.02	-0.02, 0.07	0.325
RHR ≥70bpm vs <60bpm	-0.01	-0.07, 0.04	0.632	-0.001	-0.06, 0.04	0.736
GFAP						
RHR <60bpm vs 60-69bpm	-0.01	-0.22, 0.20	0.923	-0.01	-0.22, 0.20	0.923
RHR ≥70bpm vs 60-69bpm	0.04	-0.14, 0.23	0.634	0.04	-0.14, 0.23	0.634
RHR ≥70bpm vs <60bpm	0.06	-0.16, 0.27	0.607	0.06	-0.15, 0.27	0.565

Model A: adjusted for age, sex and BMI

Model B: adjusted for age, sex, BMI, hypertension, diabetes mellitus, cognitive enhancers

BMI, body mass index; NFL, neurofilament light chain; RHR, resting heart rate
